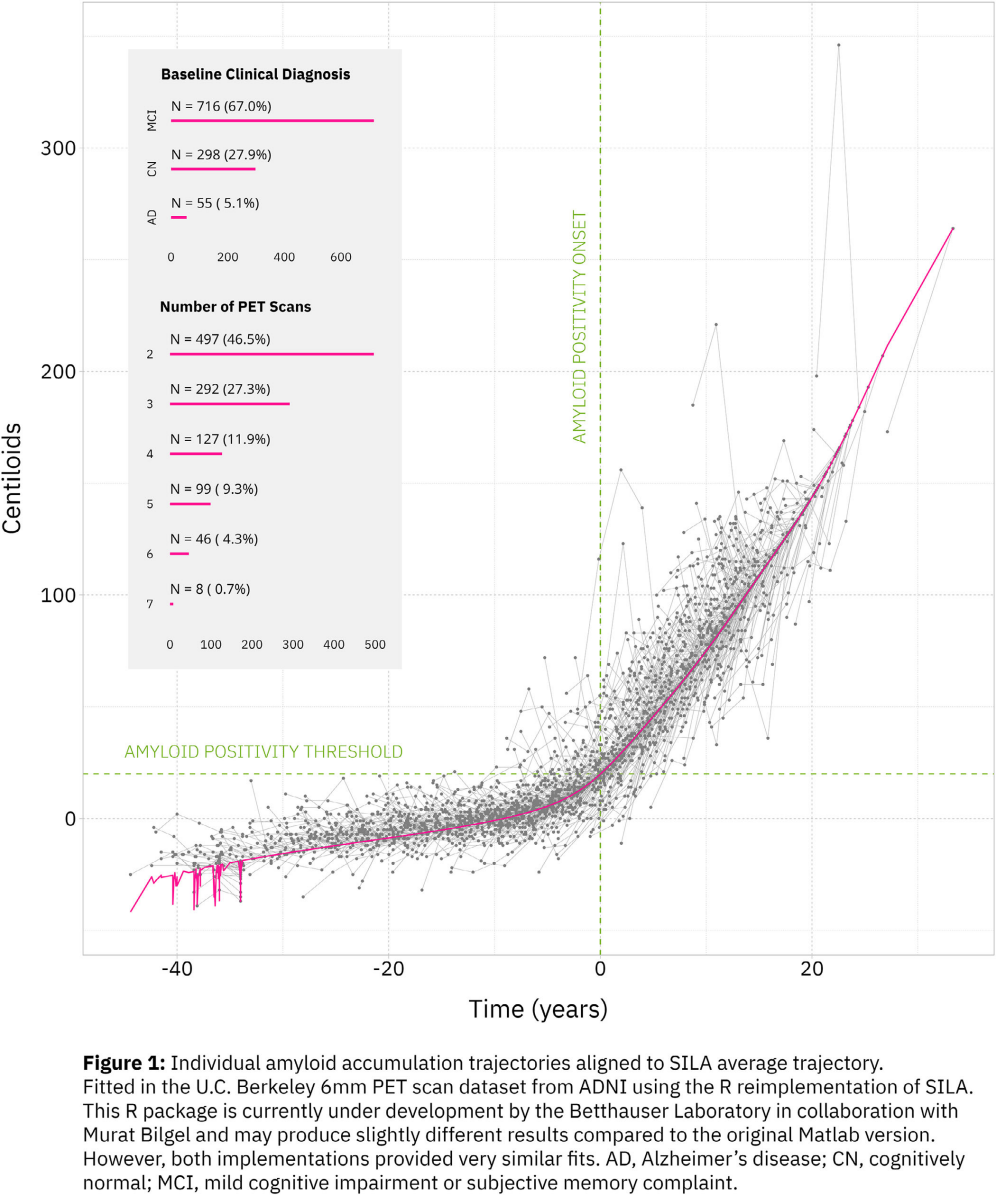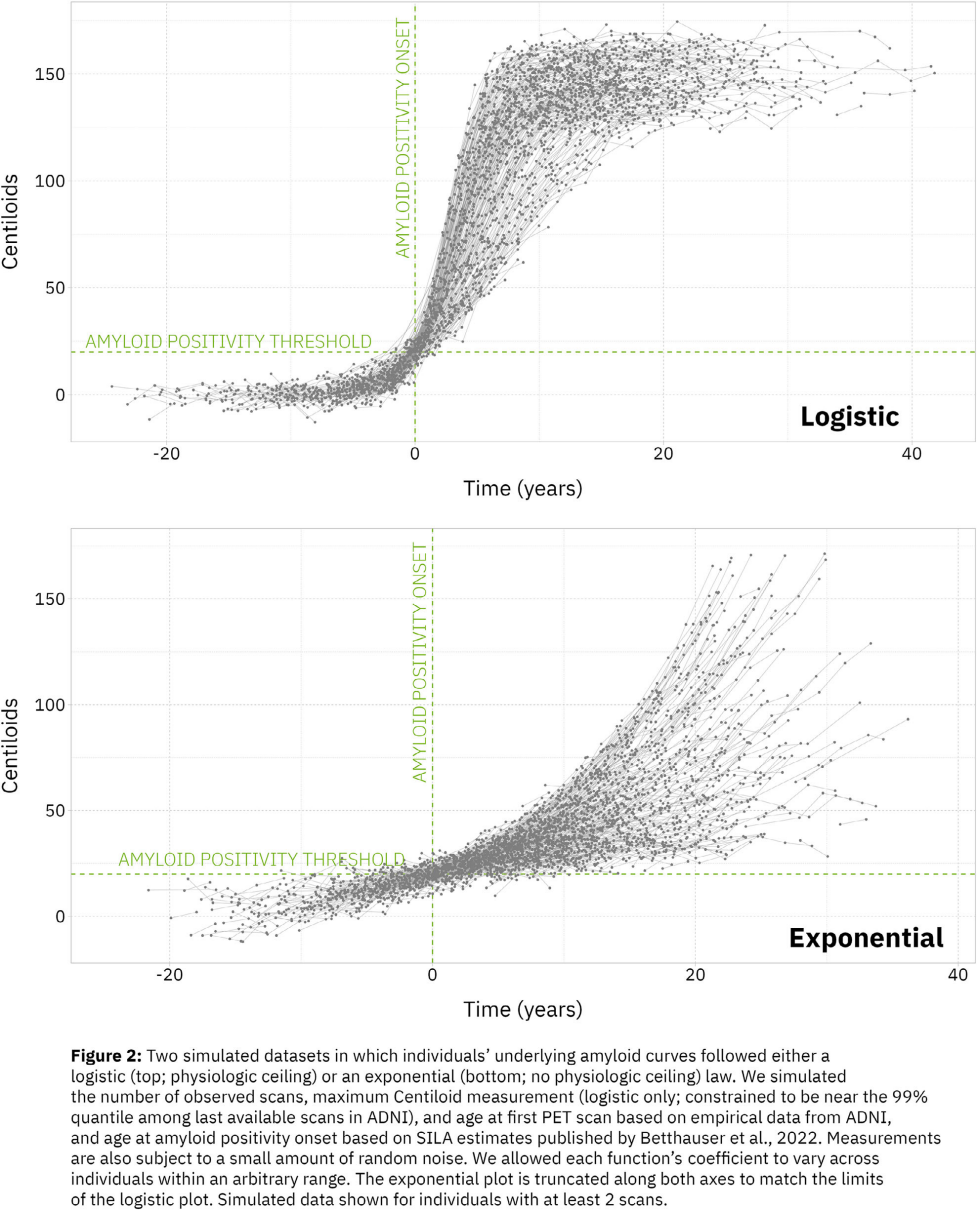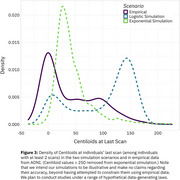# Evaluating the Presence of a Physiologic Ceiling in Amyloid Trajectories: Insights from the Sampled Iterative Local Approximation (SILA) Algorithm and Simulations

**DOI:** 10.1002/alz70862_109719

**Published:** 2025-12-23

**Authors:** Sarah F Ackley, Jason R. Gantenberg, Margo B. Heston, Renaud La Joie

**Affiliations:** ^1^ Brown University, Providence, RI USA; ^2^ University of California, San Francisco, Memory and Aging Center, San Francisco, CA USA; ^3^ Department of Neurology, University of California, San Francisco, San Francisco, CA USA

## Abstract

**Background:**

With continued collection of amyloid positron emission tomography (PET) neuroimaging and new quantitative approaches to analyze longitudinal PET data, the Alzheimer’s disease (AD) research field is now positioned to determine amyloid trajectories empirically. Previous studies proposing a physiologic ceiling generally do not evaluate the direct relationship between amyloid levels and time. However, newer studies using sampled iterative local approximation (SILA), a nonparametric algorithm that estimates trajectories with data reflecting differential scan ages/intervals, have not indicated the presence of an accumulation plateau at high amyloid burden. These findings contradict temporal models of AD development that argue for a physiologic ceiling.

**Method:**

We simulated amyloid trajectories informed by Alzheimer’s Disease Neuroimaging Initiative (ADNI) study data and the Jack model of AD pathogenesis. Empirically informed stochastic parameters included age at first PET scan, number of scans per individual, and inter‐scan intervals. Estimated age of amyloid positivity onset was drawn from a distribution based on prior published literature (Betthauser et al. 2022). Simulations assume interindividual variability in the physiologic ceiling and rates of amyloid accumulation.

**Result:**

Reimplementing SILA in ADNI shows an apparent lack of a physiologic ceiling for amyloid, consistent with prior literature (Figure 1). Simulations that assume a physiologic ceiling show qualitatively different trajectories from trajectories in ADNI data (Figure 2), and lack an increase in density at high Centiloids characteristic of individuals approaching a ceiling (Figure 3). We are developing an R package for performing realistic amyloid trajectory simulations under various assumptions about trajectory shape and variability.

**Conclusions:**

Amyloid trajectories in ADNI aligned based on SILA‐estimated age of amyloid‐positivity onset suggest an apparent lack of a physiologic ceiling for amyloid. This result is in contrast to prior studies which argue for a ceiling based on differences in amyloid by clinical stage and baseline amyloid level. Additionally, generating data based on influential models of temporal AD biomarker evolution does not produce trajectories consistent with those observed in ADNI. Growing longitudinal data availability and new quantitative tools should allow us to formally evaluate prevailing models of AD biomarker evolution.